# The combined effect of epigenetic inhibitors for LSD1 and BRD4 alters prostate cancer growth and invasion

**DOI:** 10.18632/aging.102630

**Published:** 2020-01-05

**Authors:** Jianlin Wang, Qian Yu, Zhaoping Qiu, Tao Dai, Shuxia Wang, Xiuwei Yang, B. Mark Evers, Yadi Wu

**Affiliations:** 1 Department of Pharmacology and Nutrition Science, The University of Kentucky, Lexington, KY 40506, USA; 2 Markey Cancer Center, College of Medicine, The University of Kentucky, Lexington, KY 40506, USA; 3 Institute of Clinical Medicine, The First Affiliated Hospital of University of South China, Hengyang, Hunan 421001, P.R. China; 4 Department of Surgery, The University of Kentucky, Lexington, KY 40506, USA

**Keywords:** BRD4, inhibitor, invasion, LSD1, prostate cancer

## Abstract

Epigenetic modifications play an important role in prostate tumor development and progression. Epigenetic drugs are emerging as effective modulators of gene expression that act on pathways potentially important in the control of cancer clinically. We investigated two different epigenetic modulating drugs, SP-2509 and JQ1, that target histone lysine demethylase 1 (LSD1), and bromodomain-containing protein (BRD), respectively and their combined effect in three different prostate cancer (PCa) types: 1) androgen receptor (AR)-positive and androgen-sensitive; 2) AR-positive but castration-resistant; and 3) androgen-nonresponsive. We found combined treatment provided a synergistic growth inhibition in castration-resistant PCa cells but knockdown of AR reduced sensitivity to both inhibitors in these cells. In the androgen-sensitive cell lines, AR knockdown attenuated sensitivity to the LSD1 inhibitor but not the JQ1 inhibitor. Strikingly, treatment with SP-2509 slightly, and JQ1 markedly increased invasion in PCa cells with high AR expression but decreased invasion in PCa cells with low/negative AR expression. Our results suggest that these two epigenetic drugs are novel and promising compounds for the development of PCa therapeutics, particularly for castration-resistant disease. However, due to the potential risks, including metastasis, caution must be exercised in the clinical setting.

## INTRODUCTION

Prostate cancer (PCa) is one of the most frequently diagnosed cancers and is the second leading cause of cancer deaths in men worldwide [1]. Despite the success of many therapies including surgery, radiotherapy and AR targeting therapeutics, about 20–53% of cases become resistant to conventional treatments and relapse. A large proportion of these patients develop metastatic lesions for which there is no curative treatment [2]. Therefore, an urgent need exists for the development of novel therapeutic strategies for treatment of PCa.

Recently, epigenetic modifications, including DNA methylation patterns and post-translational modification of histone tails, have emerged as significant participants in PCa progression. Since epigenetic modifications are potentially reversible, much effort has been directed toward understanding the mechanisms of epigenetic aberrations that promote cancer, and for development of new therapies to block or reverse them [3, 4]. The amine oxidase LSD1 was the first discovered H3K4 lysine-specific demethylase [5, 6]. LSD1 expression is increased significantly and was positively correlated with distant metastases and poor prognosis in PCa [7]. Inhibition of LSD1 is an effective strategy for multiple malignancies including small lung cancer and PCa [8–10]. To date, a handful of small molecular inhibitors of LSD1 have been developed [11–15]. SP-2509 is unique among LSD1 inhibitors because it recapitulates the effects of LSD1 RNAi [16]. However, it is difficult to efficiently inhibit tumor progression by targeting a single epigenetic modification. To overcome this limitation, combined inhibition of epigenetic modifiers is examined. Combination therapy, targeting different pathways or the same critical molecule but for distinct effects, may provide a more efficacious response, particular in solid tumors.

BRD4 is a conserved member of the bromodomain and extraterminal domain (BET) family of chromatin readers [17]. BRD4 protein expression at diagnosis positively associates with a poor overall survival in patients with prostate cancer, and the strength of this association increases as castration-resistant disease develops [18]. BRD4 inhibitors have shown promising activity against multiple cancers in pre-clinical studies, and at present there are five BRD4 inhibitors in phase I/II clinical trials [19–21]. JQ1 is a potent, selective small molecule inhibitor of BET bromodomains targeting BRD2,-3, -4 and the testis-specific protein BRDT with a remarkable success of BRD4 [19, 22]. JQ1 inhibits BRD4-AR binding, and results in reduced AR gene transcription and subsequent diminished AR signalling [23]. Most importantly, several studies show that JQ1 acts synergistically with other inhibitors to enhance apoptosis [24–26].

Both LSD1 and BRD4 sustain embryonic stem cell self-renewal, and control cell fate decisions by positively regulating the expression of pluripotency genes, such as Oct4 [6, 27, 28]. In addition, both LSD1 and BRD4 are highly expressed in PCa and positively associate with a poor overall survival in patient with PCa [18, 29]. Furthermore, both LSD1 and BRD4 interact with AR as a coactivator and play an important role in AR signalling, especially in AR-positive but castration-resistant PCa [23, 29, 30]. Interestingly, inhibition of LSD1 overcomes stable epigenetic resistance thus re-distributes transcriptional co-activators, including BRD4, and provides the opportunity to disable their activity and overcome epigenetic resistance [31]. Therefore, we were interested in exploring the possible benefits of using a combination of SP-2509 and JQ1 in PCa. We first examined proliferation in three different types of PCa including AR positive androgen-sensitive, AR positive but castration-resistant, and AR negative PCa cell lines treated with inhibitors of LSD1 and BRD4, alone or in combination. We show that in the AR-positive and androgen-sensitive cell lines AR expression is sensitive to LSD1 inhibition, but not to BRD4 inhibition. In contrast, loss of AR completely disrupted the suppressive effects of both LSD1 and BRD4 inhibitors in the castration-resistant PCa cells. Furthermore, we found that these two inhibitors exerted different effects on tumor metastasis in cells with distinct extent AR expression. Finally, we assessed potential mechanisms that regulate LSD1 and BRD4 activity and drive PCa growth and metastasis. Our results suggest that epigenetic inhibition presents an additional therapeutic approach for treating PCa but adverse effects related to the prostate phenotype must be considered.

## RESULTS

### SP-2509 and JQ1 display different effects on AR positive and AR-negative PCa, and have a hybrid effect on castration-resistant PCa cells

**Figure 1:**
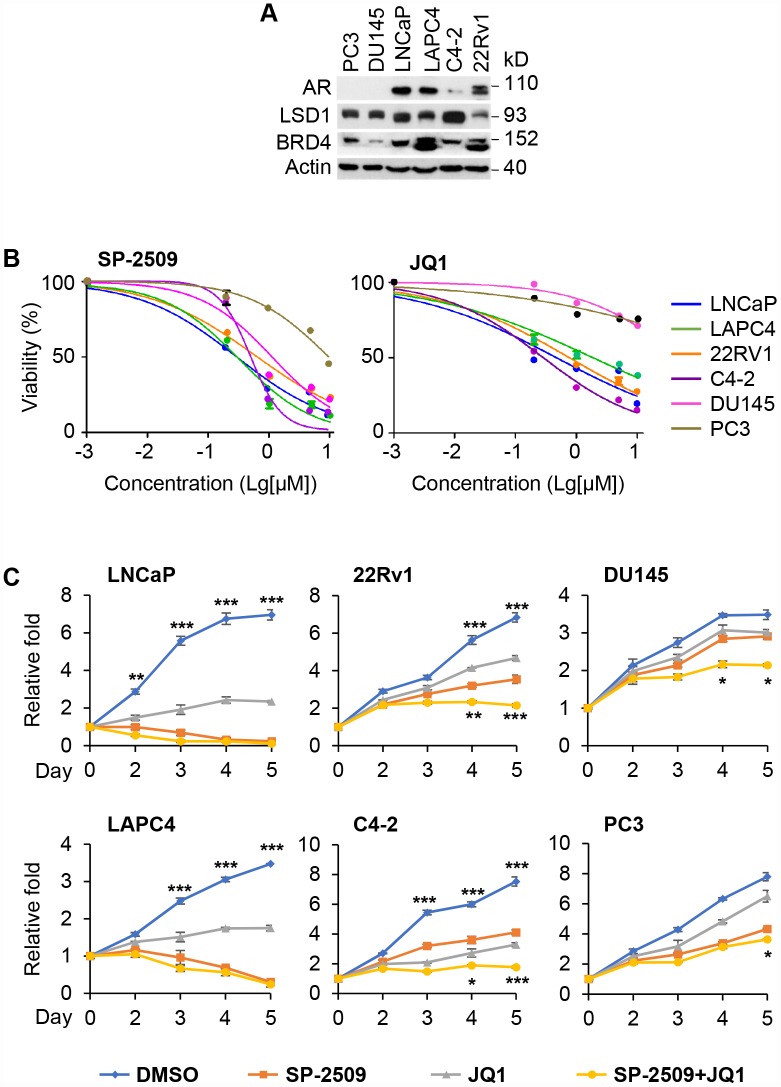
**Inhibition of LSD1 reduces the proliferation in both AR-positive and AR-negative PCa cells but inhibition of BRD4 has no effect on AR-negative PCa cells.** (**A**) Western blot analysis of AR, LSD1 and BRD4 expression in PC3, DU145, LNCaP, LAPC4, C4-2 and 22Rv1 cells. (**B**) The indicated cancer cells were treated with different doses of SP-2509 or JQ1 for 72 h and cell proliferation was determined by MTT assay. (**C**) The indicated cancer cells were treated with 1μM JQ1 or SP-2509 alone, or in combination for different time periods and cell proliferation was determined by MTT assay. Graphic data are the means ± SD of four replicate experiments. Statistical significance are determined by ANOVA with: * indicates P < 0.05; *** indicates P < 0.001, # indicates no significance.

To interrogate the combined effect of these two epigenetic inhibitors on PCa, we first examined the expression of BRD4 and LSD1 in androgen-sensitive AR-positive PCa cell lines (LNCaP and LAPC4), AR-positive but castration-resistant cell lines (22Rv1 and C4-2) and AR negative prostate cell lines (PC3 and DU145) (Figure 1A). AR levels in LNCaP, LAPC4 and 22Rv1 cells are high and similar with low expression in C4-2 cells (Figure 1A) [32]. All six PCa cell lines expressed high levels of BRD4 and LSD1. We then treated these cells with SP-2509 and JQ1, respectively. A dose-dependent decrease in cell viability was observed after 72 h of treatment with SP-2509 in all PCa cell lines with the 1μM treatment providing more than 50% loss of cell viability in most of these cells (Figure 1B). Therefore, we used this dose in our later studies. However, treatment with JQ1 induced a dose-dependent decrease in cell viability in AR-positive but not AR-negative prostate cells. We then examined the effects of treating these cells with JQ1 and SP-2509, alone or combination. In LNCaP and LAPC4 cells, treatment with SP-2509 dramatically inhibited cell growth while JQ1 treatment led to a more modest growth inhibition. Treatment with both JQ1and SP-2509 provided no additional growth inhibition in LNCaP and LAPC4 cells over SP-2509 alone (Figure 1C, left panels). Intriguingly, the effect of these compounds was significant in castration-resistant cell lines (22Rv1 and C4-2) (Figure 1C, middle panels); strikingly, the combined treatment had an additional effect on growth inhibition in these two castration-resistant cells. In contrast, treatment of PC3 and DU145 cells with JQ1 showed no significant inhibition in cell growth. Exposure to SP-2509 modestly blocked cell growth and co-treatment with JQ1 and SP-2509 also led to a similar modest reduction in cell viability and no additive effect over SP-2509 alone (Figure 1C, right panels).

**Figure 2:**
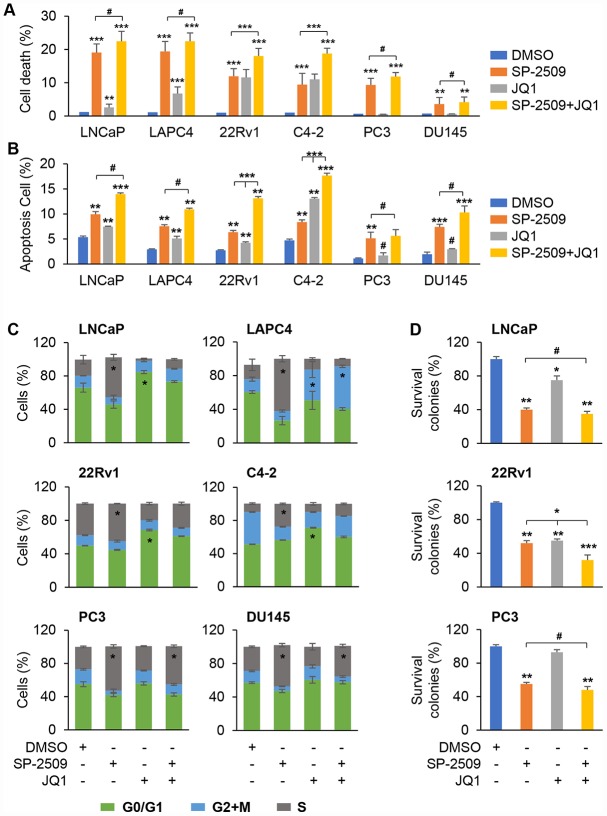
**Inhibition of LSD1 induces cell apoptosis and arrests cells in S phase but inhibition of BRD4 exhibits no apoptotic effect in PC3 and DU145 cells.** (**A**–**B**) Analysis of apoptosis in LNCaP, LAPC4, 22Rv1, C4-2, PC3 and DU145 cells after 72 h treatment with 1 μM JQ1 or SP-2509 alone, or in combination. Cell death was assessed using propidium iodide (PI) staining (**A**) or Apoptosis was assessed by Annexin-V and PI staining followed by FACS analysis (**B**). (**C**) Graphic representation of cell cycle distribution for LNCaP, LAPC4, 22Rv1, C4-2, PC3 and DU145 cells after 72 h treatment with 1 μM JQ1 or SP-2509 alone, or in combination. Duration of each cell cycle stage was assessed using PI staining followed by FACS analysis. (**D**) Colony formation for cells after 72 h treatment with 1 μM JQ1 or SP-2509 alone, or in combination. Graphic data are the means ± SD of four replicate experiments. Statistical significance are determined by ANOVA with: * indicates P < 0.05; ** indicates P < 0.01; *** indicates P < 0.001, # indicates no significance.

To explore the growth inhibition induced by JQ1 and SP-2509, we evaluated apoptosis using two different techniques, and cell cycle intervals after treatment in these three different PCa cell types. Consistent with results obtained with cell proliferation, treatment of LNCaP and LAPC4 cells with SP-2509 resulted in a marked increase in apoptosis compared with vehicle treatment (Figure 2A and Supplementary Figure 1A), but the effect of JQ1 treatment on apoptosis was much less dramatic. No additional increase in apoptosis was observed in LNCaP and LAPC4 cells when JQ1 was added to SP-2509. Consistent with our previous results, both SP-2509 and JQ1 resulted in a marked increase in apoptosis and had an additive effect in the castration-resistant cells, 22Rv1 and C4-2. In agreement with the cell proliferation finding for PC3 and DU145 cells, SP-2509 but not JQ1 induced cell death, and co-treatment with JQ1 and SP-2509 provided no additional cell death over that observed with SP-2509 alone (Figure 2A and Supplementary Figure 1A). To confirm these cell death findings, we performed the apoptosis assay with Annexin V-FITC/PI flow cytometry. Again, SP-2509 significantly induced apoptotic cell death in all these three PCa cells types while JQ1 induced apoptotic cell death only in AR positive cells but not in AR-negative cells (Figure 2B and Supplementary Figure 1B). Notably, combined treatment with SP-2509 and JQ1 had an additive effect in castration resistant prostate cells (22Rv1 and C4-2) but not in other two type of PCa cells. We also found that SP-2509 treatment led to a significant number of cells at the S phase in all the PCa cell types while the JQ1 treatment led to an accumulation in the G0/G1 phase in LNCaP, 22Rv1 and C4-2 cells (Figure 2C). We did not detect any significant change in cell cycle pattern for DU145 and PC3 cells treated with JQ1 alone, but noted an accumulation of cells in S phase with co-treatment as well as with SP-2509 alone (Figure 2C). We also assessed colony formation to investigate a long-term effect of JQ1 and SP-2509 on proliferation. As shown in Figure 2D, the colony formation for all cell lines was reduced after exposure to SP-2509. Furthermore, the reduction was more apparent when SP-2509 was combined with JQ1 in the castration-resistant cells. However, JQ1 significantly inhibited colony formation only in the AR-positive cells and had no significant effect on AR-negative cells. These results indicate that the SP-2509 and JQ1 have different effects in AR-positive and AR-negative PCa cells.

### AR is critical for LSD1 inhibition

**Figure 3:**
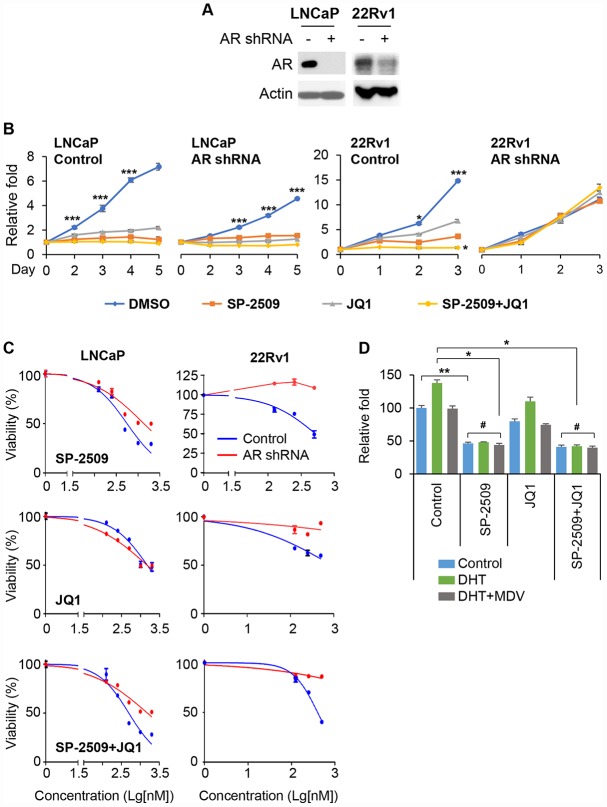
**Knockdown of AR reduces LSD1 inhibition.** (**A**) Immunoblot confirming knockdown of the AR in LNCaP and 22Rv1 cells. (**B**) Graphic representation of control and stable AR shRNA knockdown LNCaP and 22Rv1 cells treated with 1μM JQ1 or SP-2509 alone, or in combination for different time periods. Cell proliferation was determined by MTT assay. (**C**) Graphic representation of control and stable AR shRNA knockdown cells treated with increasing concentrations of SP-2509 or JQ1 alone, or in combination for 48 h; cell proliferation was determined by MTT assay. (**D**) LNCaP cells were treated with 1μM SP-2509 or JQ1 alone, or in combination with DHT or/and enzalutamide (MDV) for 72 h. Statistical difference are determined by ANOVA with: * indicates P < 0.05; ** indicates P < 0.01; *** indicates P < 0.001, # indicates no significance.

Because both BRD4 and LSD1 interact with the AR and are recruited to AR target genes, and because JQ1 and SP-2509 treatments lead to different effects in AR-positive and AR-negative cells, we next sought to investigate whether the drug-induced growth inhibitions were associated with disturbance in AR expression. To test this concept, we first infected LNCaP (AR^+^ and responsive) and 22Rv1 cells (AR^+^ and non-responsive) with validated AR shRNA lentivirus, and achieved almost complete depletion of AR expression (Figure 3A). We observed that depletion of AR significantly reduced the sensitivity of LNCaP cells to SP-2509 but not JQ1 (Figure 3B). Surprisingly, AR knockdown completely abolished the inhibition produced by SP-2509 or/and JQ1 in 22Rv1 cells. To confirm these observations, we treated the cells with different concentrations of JQ1 or SP-2509 for 48 h. We found that SP-2509 had a less pronounced effect on proliferation in AR-knockdown cells compared to the control in LNCaP cells (Figure 3C). However, there was no difference in growth inhibition between control and AR-knockdown cells with JQ1 treatment. And again, AR depletion resulted in a refractory response to SP-2509 or/and JQ1 treatment in 22Rv1 cells. We also evaluated the effect of these two inhibitors in LNCaP cells treated with dihydrotestosterone (DHT), the active androgen metabolite, for 48 h. Corroborating other reports, we found that DHT-treatment significantly increased LNCaP cell proliferation (Figure 3D); however, SP-2509 abolished the proliferation induced by DHT. In addition, we treated the LNCaP cells with SP-2509 in combination with DHT-enzalutamide (MDV), a next-generation AR antagonist. Treatment with SP-2509 was more effective compared with treatment with DHT-enzalutamide in inhibiting cell proliferation. However, combinational treatment with SP-2509 and DHT-enzalutamide had no additional effect compared to SP-2509 treatment alone. JQ1 had only a modest effect on cell proliferation under these conditions.

**Figure 4:**
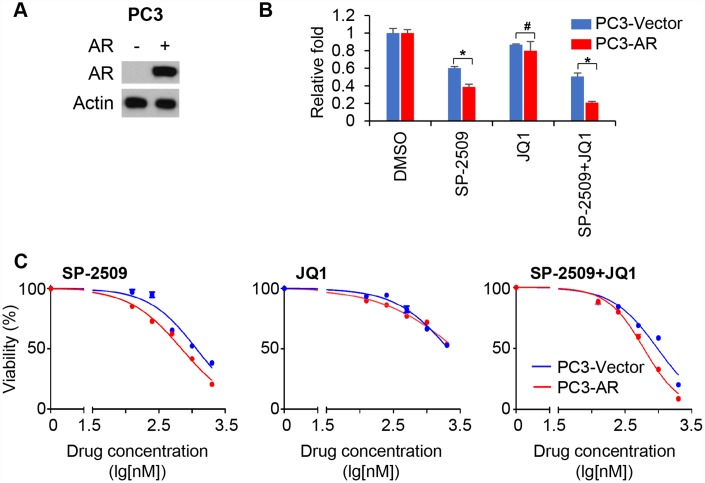
**Expression of AR sensitizes AR-negative cells to LSD1 inhibition.** (**A**) Immunoblot confirming ectopic expression of the AR in PC3 cells. (**B**) Graphic representation of control and stable AR-expressing PC3 treated with 1μM JQ1 or SP-2509 alone, or in combination for 72 hr. Cell proliferation was determined by MTT assay. (**C**) Graphic representation of control and stable AR-expressing PC3 cells treated with increasing concentrations of SP-2509 or JQ1 alone, or in combination for 48 h and cell proliferation were determined by MTT assay. Statistical difference are determined by ANOVA with: * indicates P < 0.05; # indicates no significance.

To corroborate the role of AR expression in the action of two inhibitors, we ectopically expressed AR in PC3 cells (Figure 4A). Proliferation analysis demonstrated that AR-overexpressing PC3 cells were more sensitive to SP-2509 compared with control cells (Figure 4B). However, AR expression produced no inhibition in cell proliferation by JQ1. Treatment with different concentrations of JQ1 or SP-2509 confirmed that AR overexpression sensitized SP-2509-induced growth inhibition but not JQ1-induced growth inhibition in PC3 cells (Figure 4C). Again, treatment with SP-2509 and JQ1 had no additive effect on decreasing cell viability compared to SP-2509 treatment alone in AR-overexpressing PC3 cells. Together, these results suggested that AR expression is critical for mediating control of cell proliferation in AR-positive/androgen-sensitive PCa cell lines; inhibition of LSD1 function by SP-2509 decreased proliferation in these cells while inhibition of BRD4 had little effect. However, AR expression is crucial for both of these inhibitors in AR-positive but castration-resistant PCa cells.

### Inhibition of LSD1 and BRD4 enhances invasion in high AR-expressing PCa cells but impairs invasion in low/negative AR-expressing PCa cells

**Figure 5:**
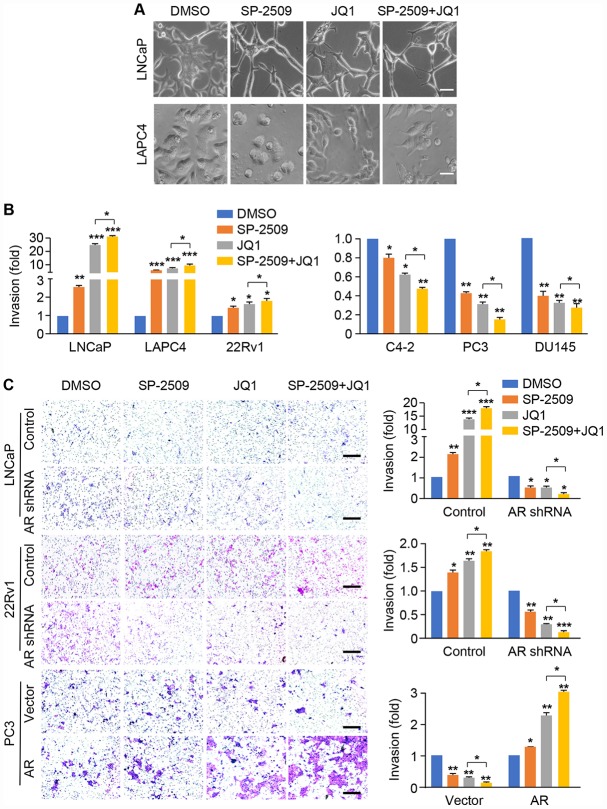
**SP-2509 and JQ1 increase cell invasion in high AR-expressing cells (LNCaP, LAPC4 and 22Rv1) but decrease cell invasion in low/negative AR-expressing cells (C4-2, PC3 and DU145).** (**A**) Cell morphology for LNCaP and LAPC4 cells was shown after treatment with 1μM SP-2509 or JQ1 alone, or in combination for 24 h. (**B**) Graphic representation of the fold change in invading cells with statistical significance presented for AR-positive and AR-negative cell types. (**C**) The cell invasion analysis was performed in AR knockdown LNCaP and 22Rv1 cells, or AR expressing PC3 cells with treatments as described in (A). Representative images are shown (left) and graphic representation of the fold changes in the invading cells with statistical significance was indicated (right). Statistical differences are determined by ANOVA with: * indicates P < 0.05; ** indicates by P < 0.01; *** indicates P < 0.001. Scare bar, 200μm.

To investigate the role of these two inhibitors on metastatic capability, we treated LNCaP and LAPC4 cells with SP-2509 and JQ1 alone or combination for 24 h; we noticed that the cells acquired a spindle-shaped morphology (Figure 5A). To assess whether these morphologic changes were associated with invasive capability, we evaluated the effects of JQ1 and SP-2509 on tumor invasiveness for AR-positive and AR-negative cell types. Cells were pre-treated with JQ1 and SP-2509 alone or in combination for 24 h and then examined for invasive activity through a Matrigel-coated membrane. As shown in Figure 5B and Supplementary Figure 2, JQ1 treatment dramatically increased cell invasion in LNCaP and LAPC4 cells, with only a slight increase in invasive capacity after SP-2509 treatment. In addition, the combined treatment of JQ1 and SP-2509 increased invasion compared to either treatment alone (Figure 5B). Strikingly, we found that JQ1 and SP-2509 appear to have different effects in castration-resistant cells. Both SP-2509 and JQ1 increased invasion in 22Rv1 cells but inhibited invasion in the C4-2 cells. In contrast, both of SP-2509 and JQ1 significantly inhibited tumor cell invasion in PC3 and DU145 cells; co-treatment with JQ1 and SP-2509 synergistically reduced cell invasion in PC3 and DU145 cells. To further explore the effect of AR expression on cell invasion, we treated the LNCaP AR-knockdown cells, 22Rv1 AR-knockdown cells or PC3 AR-expressing cells with SP-2509 and JQ1 alone or in combination for 24 h. Strikingly, depletion or expression of AR completely reversed the effect of SP-2509 and JQ1 on these cells (Figure 5C). Both JQ1 and SP-2509 suppressed invasion in the modified LNCaP and 22Rv1 AR-knockdown cells, while these inhibitors promoted invasion in AR-expressing PC3 cells. These results suggest that JQ promotes a robust increase in tumor invasive capacity in high AR-expressing PCa cells but decreases invasive capacity in low/negative AR-expressing PCa cells and that the two compounds act in a synergic manner. Most importantly, AR expression plays a key role in the inhibition of invasion with JQ1 and SP-2509.

### LSD1 and BRD4 inhibition have different effects on target genes in androgen-dependent and androgen-independent PCa cells

**Figure 6:**
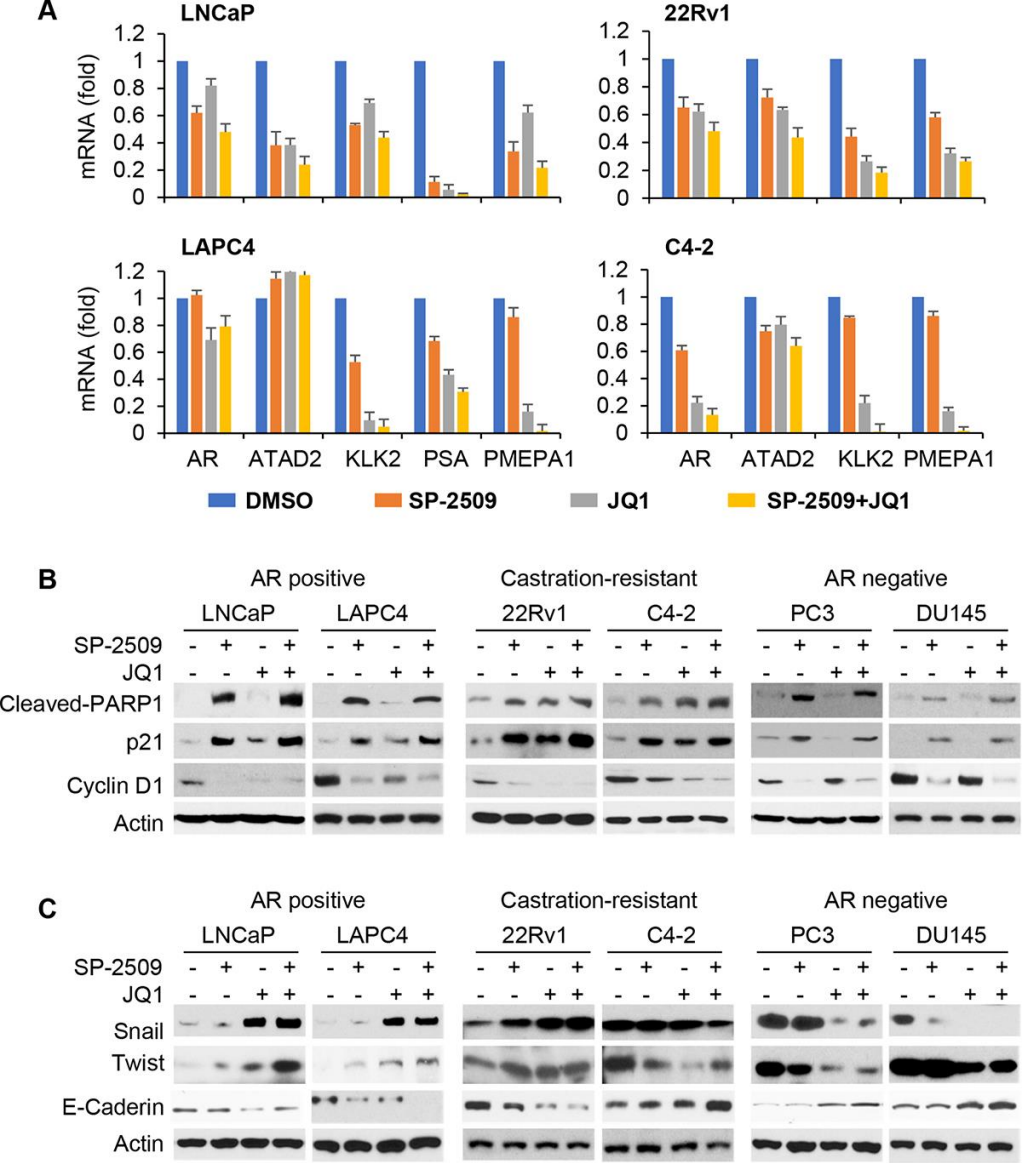
**LSD1 and BRD4 inhibition have different target genes in AR-positive and AR-negative PCa cells.** (**A**) Cells were treated with 1 μM JQ1 or 1 μM SP-2509 alone, or in combination for 72 h. mRNA expression of selected AR target genes was measured by real-time PCR assay. Data were reported as the fold change in mean levels ±SD. (**B**–**C**) Cells were treated with 1 μM JQ1 or 1 μM SP-2509 alone, or in combination for 72 h. Total protein lysates were analyzed by immunoblot using the indicated antibodies.

Our results suggested that the AR was important for both LSD1 and BRD4 inhibition. To further examine these relationship, we first knocked down LSD1 or BRD4 and assessed AR expression in LNCaP and 22Rv1 cells. Silencing LSD1 or BRD4 not only reduced AR full length expression in LNCaP and 22Rv1 cells but also AR-V7 in 22Rv1 cells (Supplementary Figure 3A). Consistent with this, treatment with SP-2509 or JQ1 recapitulated the results (Supplementary Figure 3B). Combined treatment with SP-2509 and JQ1 further reduced the AR expression. We then evaluated mRNA expression levels of AR-target genes using real-time qPCR. We found that treatment with the two inhibitors decreased AR expression and classical AR-target genes (*ATATD2, KLK2, PSA* and *PMEPA1*) in all AR-positive prostate cell lines (Figure 6A). In support of our data on cytotoxic effects, treatment with SP-2509 was associated with a remarkable increase in cleaved PARP in all three type PCa cells while treatment with JQ1 slightly increased expression of cleaved PARP (Figure 6B) in AR-positive but not AR-negative PCa cells. Importantly, both SP-2509 and JQ1 increased cleaved PARP expression in castration-resistant PCa cells. Previous findings demonstrated that LSD1 inhibition blocks neuroblastoma cell proliferation and regulates pivotal genes controlling the cell cycle such as CDKN1A/p21 [33]. Therefore, we examined the expression of genes controlling cell cycles. Treatment with SP-2509 in AR-positive (LNCaP, LAPC4, 22Rv1 and C4-2) prostate cells significantly increased expression of p21 but decreased expression of Cyclin D1 (Figure 6B). Consistent with apoptotic results, JQ1 only slightly increased expression of p21 and down-regulated expression of Cyclin D1 in LNCaP and LAPC4 cells but significantly regulated these two genes in 22Rv1 and C4-2 cells. In DU145 and PC3 cells, the expression levels of these genes were unaffected by JQ1 treatment but SP-2509 treatment result in a slight increase in p21 and decrease in Cyclin D1. These results are agreement with our previous findings that SP-2509 inhibited cell growth and induced cell apoptosis in all these cells, and that JQ1 induced cell apoptosis only in AR-positive cells but not in AR-negative cells.

In human PCa, upregulation of Twist was positively correlated with Gleason grades, cell migration and invasive capability [34]. We previously found that Twist interacts with BRD4 [35]. To provide information on the potential molecular mechanism regarding the divergent invasive responses to SP-2509 and JQ1 in these three different types of PCa cells, we examined Twist and Snail expression (Figure 6C). Surprisingly, we found that JQ1 markedly increased Twist and Snail expression while decreasing E-Cadherin expression in high AR-expressing prostate cells (LNCaP, LAPC4 and 22Rv1). However, treatment with either SP-2509 or JQ1 decreased Twist and Snail expression and increased E-Cadherin expression in low/negative AR-expressing prostate cells (C4-2, PC3 and DU145). In addition, SP-2509 acted in concert with JQ1 to reduce Twist and Snail expression. These results are consistent with the differing invasive responses to SP-2509 and JQ1.

### LSD1 and BRD4 inhibitors inhibit tumor growth but the BRD4 inhibitor increases tumor metastasis *in vivo*

**Figure 7:**
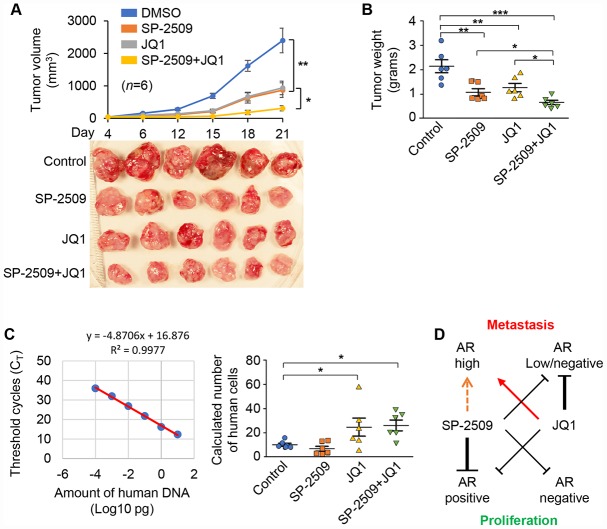
**SP-2509 and JQ1 inhibit tumor growth but JQ1 increase tumor metastasis *in vivo*.** (**A**) Tumor growth of 22Rv1 xenografts was measured. Tumor volume (upper) and tumors harvested at the end time point (Day 21) from these mice (lower) are shown. Graphic data are presented as the mean ±SD. (**B**) The mean of tumor weight from (A) at the end time point (Day 21) was shown. (**C**) Standard curve for detection of human genomic DNA by Alu-qPCR (left) and detection of human cells in mouse femur from (A) by Alu-qPCR (right). (**D**) A model of LSD1 and BRD4 inhibition in PCa. Statistical differences are determined by ANOVA with: * indicates P < 0.05; ** indicates P < 0.01.

To assess the effects of these two inhibitors *in vivo*, we implanted the 22Rv1 cells into nude mice treated with SP-2509 and JQ1 alone or together. As shown in Figure 7A, monotherapies with SP-2509 or JQ1 significantly inhibited the tumor growth compare with untreated control group. Importantly, the combination of SP-2509 and JQ1 led to a significantly greater inhibition in tumor growth than did either SP-2509 or JQ1 alone. In addition, the tumor weights were also significantly reduced after drug treatment (Figure 7B). To investigate whether SP-2509 and JQ1 treatment alter spontaneous metastasis in our 22Rv1 xenograft model, we isolated femur and liver from drug-treated mice and found evidence of disseminated cells in the femur but not in the liver after JQ1 treatment (Figure 7C and data not shown). However, SP-2509-treated mice showed no evidence of metastasis. In summary, these studies support the notion that JQ1 inhibits tumor growth but induces metastasis in high AR-expressing PCa cells. These results are also consistent with our observation in the cell-based study, providing additional evidence of inhibition by SP-2509 and JQ1 in PCa proliferation and metastasis *in vitro* and *in vivo.*

## DISCUSSION

Using PCa cell lines that differ in their androgen growth-dependence, we evaluated the combined action of two selective inhibitors SP-2509 and JQ1, that target the important epigenetic modifying proteins LSD1 and BRD4, respectively. The studies were initiated with the rational that combined treatment with two different epigenetic activity may provide therapeutic efficacy. We found that SP-2509 inhibited cell growth in all PCa cells and suppressed cell invasive ability in prostate cells with low or absent expression of the androgen receptor (Figure 7D). In contrast, JQ1 only inhibited cell growth in AR-positive but not AR- low/negative PCa cells. Strikingly, JQ1 markedly enhanced cell invasion in high AR-expression PCa cells but reduced cell invasion in AR low/negative PCa cells (Figure 7D). Most importantly, we found JQ1 and SP-2509 have a synergistic effect on growth inhibition only in castration-resistant PCa cells.

LSD1 interacts with AR and promotes AR-targeted genes by depressing histone marks [36]. The development of LSD1 inhibitory compounds represents a new strategy to block the activity of AR-associated PCa. In our study, SP-2509 diminished cell proliferation in all prostate tumor cells but was most dramatic in AR-positive tumor cells. This finding suggests that the LSD1 inhibitor suppresses PCa proliferation predominantly through AR associated genes. Indeed, we found that most of AR associated genes were suppressed with SP-2509 treatment (Figure 6A). Knockdown of the AR confirmed that AR expression is critical to modulate LSD1 activity. However, we also found that LSD1 suppression with SP-2509 treatment reduced cell viability in AR-null PCa cells, which is consistent with previous reports [16]. In addition, knockdown of AR did not completely abolished the effect of SP-2509 treatment in LNCaP cells (Figure 3B), which suggests an important AR-independent role of LSD1 in prostate cancer progression [16]. It is noteworthy that we did not stimulate cells with high doses of supplemental androgens when conducting experiments to examine the effect of AR activity on gene-expression changes after JQ1 or SP-2509 treatment. Therefore, we cannot rule out the possibility that additional genes may be modulated under high-androgen conditions.

AR regulation is implicated in response to BET inhibition, and high AR-expressing prostate cells were preferentially sensitive to JQ1 treatment [37, 38]. Consistent with a previous report showing that knockdown of BRD4 decreased viability in the AR-positive but not AR-negative cell lines [37, 39], we found that only AR-positive cells were sensitive to JQ1-induced apoptosis and cell cycles arrest in G1 phase; we did not find a significant effect on the growth in AR-negative PCa cells treated with JQ1. It was reported that JQ1 inhibits PCa cell growth at least in part through MYC and AR suppression [40]. MYC signaling is an oncogenic driver for PCa progression and is a potential biomarkers for targeting BET proteins [39]. JQ1 reduced MYC levels only in AR-positive PCa cells but not PC3 and DU145 cells [41]. Maintenance of MYC expression confers de novo resistance to JQ1. Conversely, SP-2509 decreased MYC protein levels in PC3 and DU145 cells [42]. Because MYC and AR signaling are essential for prostate cancer initiation, MYC may be another key determinant both of BET bromodomain inhibitor and LSD1 inhibitor sensitivity in PCa.

It was known that the mutually exclusive expression of AR and EMT transcription factors occurs in castration-sensitive (LNCaP) and castratio-resistant (22RV1) PCa cell lines [43, 44]. In addition, up-regulation of EMT transcription factors was observed in AR-silenced cells. Conversely, AR overexpression suppresses the EMT phenotype and AR directly represses Snail [45, 46]. Strikingly, we found that JQ1 dramatically reduced cell invasion in negative/low AR-expressing cells, but increased invasion in high AR-expressing cell lines. This result was surprising. We also observed that JQ1 or/and SP-2509 treatment down-regulated AR expression (Supplementary Figure 3B). In addition, depletion or expression of AR reversed the effect of SP-2509 or/and JQ1 treatment (Figure 5). Finally, we found that Twist and Snail expression were increased with SP-2509 or/and JQ1 treatment in AR-expressing cell lines (Figure 6). These data suggested that JQ1 and SP-2509 treatment increased the expression of Twist and Snail by blocking AR signaling pathway. We knocked down LSD1 and BRD4 to determine whether depletion of either protein would increase cell invasion in high AR-expressing cells. However, our findings with SP-2509 or JQ1 treatment were not recapitulated with BRD4 or LSD1 knockdown (data not shown). It is possible that: 1) knockdown of LSD1 and BRD4 dramatically reduces the growth rate so that cell invasion could not be seen under standard conditions, three days. However, treatments with SP-2509 or JQ1 were only 24 hr; 2) knockdown of LSD1 disrupts the LSD1-containing protein complex while SP-2509 treatment only reduces the interaction between LSD1 and the corepressor for element-1-silencing transcription factor (CoREST) [47]. JQ1 is a pan-BRD inhibitors targeting BET family while knockdown of BRD4 only impaired its own function. BRD4 also has bromodomain-independent effects [48]; 3) we can not exclude any non-specific target effect of SP-2509 and BRD4.

In summary, we provide strong evidence that inhibitors of LSD1 and BRD4 effectively inhibit tumor cell proliferation in high AR-expressing PCa tumors but also increase invasion in this population of PCa cells. In contrast, treatment with JQ1 alone has no effect on tumor growth, but dramatically attenuates invasion in AR-negative PCa. Interestingly, combined treatment with SP-2509 and JQ1 synergistically inhibits growth in castration-resistant PCa cells and inhibits tumor invasion in low/negative AR-expressing PCa. Our results suggest that epigenetic inhibitors are effective in PCa and that their combination provides insight and promise for the treatment of PCa. However, it is also imperative to consider the divergent effects in prostate tumors that are heterogeneous with respect to androgen dependence.

## MATERIALS AND METHODS

### Plasmids and reagents

Validated AR shRNA lenti-virus expression plasmid and the AR ectopic expression plasmid were kindly provided by Dr. Huang (Mayo Clinic). Anti-Actin was from Sigma-Aldrich (St. Louis, MO); antibodies for AR (sc-7305), Twist (sc-15393), BRD4 (sc-48772), p21(sc-397,) and Cyclin D1(sc-717) were purchased from Santa Cruz Biotechnology (Santa Cruz, CA). Anti-LSD1 (4064), Anti-cleaved PARP (5625) and Anti-Snail (4719) were from Cell Signaling Technology (Danvers, MA). JQ1, SP-2509 and enzalutamide were from Selleckchem (Houston, TX).

### Cell culture and treatments

The human PCa cell lines, LNCaP, LAPC4, 22Rv1, C4-2, PC3 and DU145 were purchased from the American Type Culture Collection (Manassas, VA) and grown in RPMI medium plus 10% fetal bovine serum. All cell lines are tested for mycoplasma contamination regularly. As indicated, cells were treated with drugs or DMSO-containing vehicle that was equal volume to 1 μM SP-2509 or 1 μM JQ1 alone, or in combination for different time intervals, or for 3 days at different concentrations.

### Cell proliferation assay

Cell proliferation was measured using MTT assays in 96-well microplates. PCa cells were seeded in 96-well plates in RPMI1640 medium containing 10% FBS. After 24 hours incubation, the cells were treated with appropriate concentrations of drugs. Following incubation for different time intervals, 3-(4,5-dimethylthiazol-2-yl)-2,5-diphenyltetrazolium bromide (MTT) was added and the assay performed [49].

### Colony formation assay

Cells were seeded in 6-well plates and cultured in medium alone or containing different drugs for 3 days. The media were replaced without drugs. After 8 days of culture, cells were fixed in 10% formalin and stained with 0.5% crystal violet and colony numbers were counted.

### Invasion assay

Invasion assays were performed in Boyden chambers coated with Matrigel as instructed by the manufacturer (BD biosciences, San Jose, CA). The PCa cells were pretreated with inhibitor for 24 h. The cells were then seeded on the top of a Matrigel-coated membrane in the upper chamber with serum-free culture medium, while the bottom chambers was filled with culture medium supplemented with 10% FBS. After 24 h or 72 h, cancer cells on the lower side of chamber membrane were stained with crystal violent. All experiments were performed in triplicate.

### Real-time PCR

Total RNA was purified after treatment with 1 μM SP-2509 or 1 μM JQ1 alone, or in combination for 72 h. Total RNA (1μg) was reverse-transcribed into cDNA using a Superscript II First-Strand Synthesis System for RT-PCR (Invitrogen). qPCR was performed using a CFX96 Real-Time System and SYBR Green (Applied Biosystems, Foster City, CA, USA), according to the manufacturer’s instructions.

### Western blot analysis

For protein extraction, cells were washed with cold PBS and harvested by scraping into 150 μl of RIPA buffer. SDS-PAGE and western blot analysis were performed as described [50]. Membranes were incubated with specific antibodies in dilution buffer (3% BSA in TBS) overnight at 4 °C and HRP-conjugated anti-rabbit IgG at room temperature for 1 h. Antibody binding was detected using an enhanced ECL (Pierce/ThermoFisher, Waltham, MA) following manufacturer’s instructions and visualized by autoradiography with Hyperfilm.

### Flow cytometry analysis

The PCa cells were treated with inhibitors as indicated. Cell suspensions were prepared and stained with propidium iodide. Apoptosis analysis and cell cycle phase distribution were determined using the Cyto software.

### Annexin V apoptosis detection assay

The PCa cells were treated with inhibitors as indicated. Cell suspensions were used for cell apoptosis analysis by initially staining the cells with Annexin V and propidium iodide solution followed by flow cytometry analysis

### 22Rv1-Derived murine xenograft model

22Rv1 cells (3×10^6^ cells per mouse) were suspended in 100ul of PBS with 50% Matrigel (BD Biosciences) and subcutaneously inoculated into the right flank of nude male mice (Taconic, 6 weeks). Five days later, animals were randomized for control, SP-2509 alone, JQ1 alone, or the combination treatment with 6 mice each, respectively. Animals were injected intraperitoneally with SP-2509 and JQ1 every day for 3 weeks. Tumor growth was measured with digital calipers and tumor volumes were estimated from the formula: V = L×W^2^/2 (V, mm3; L, mm; W, mm). Studies were terminated by animal sacrifice; tumors were harvested and weighed. In addition, femur bone marrow and liver were harvested to determine spontaneous metastasis by measuring human Alu sequence. Briefly, genomic DNA from femur bone marrow and liver were prepared using Puregene DNA purification system (Qiagen), following by quantification of human Alu sequence by human Alu-specific fluorescent reporter-TaqMan qPCR probes as described previously [51]. All procedures for the animal study were approved by the Institutional Animal Care and Use Committee of the University of Kentucky College of Medicine and conform to the legal mandates and federal guidelines for the care and maintenance of laboratory animals.

### Statistical analysis

Each experiment was repeated three times and data were expressed as mean ± standard deviation (SD). Comparisons between two groups and among multiple groups were performed with student’s t-test and one way ANOVA, respectively. SPSS 17.0 was used for all statistical analyses.

## Supplementary Material

Supplementary Figures
