# Supplementary material for: The combined effect of epigenetic inhibitors for LSD1 and BRD4 alters prostate cancer growth and invasion
Source: Aging (Albany NY). 2020 Jan 5;12(1):397–415. doi: 10.18632/aging.102630 (PMC6977660; doi:10.18632/aging.102630)
Supplement: Supplementary Figures [file aging-12-102630-s001..pdf]

SUPPLEMENTARY FIGURES

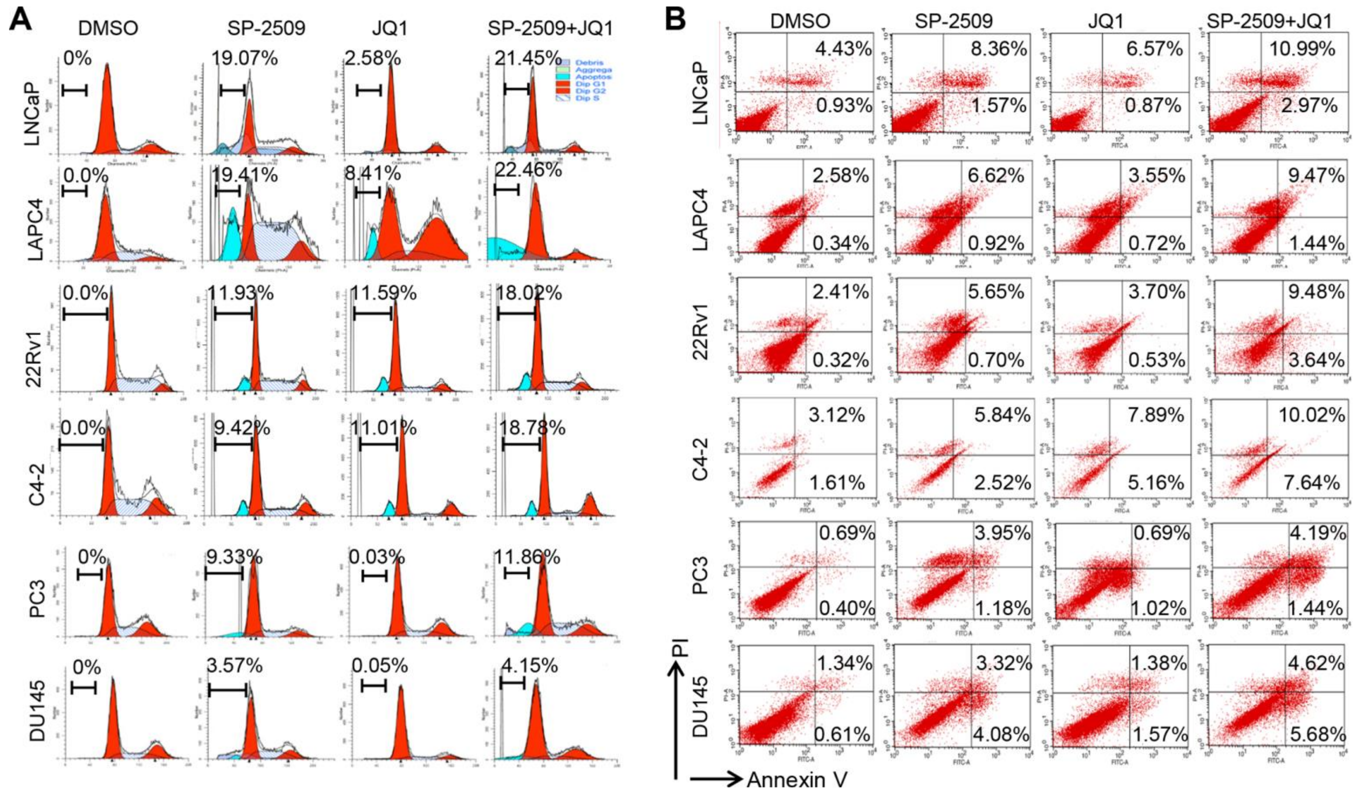

Supplementary Figure 1. Inhibition of LSD1 and BRD4 has different effects on three subtypes of Prostate Cancer. Cell death analysis in LNCaP, LAPC4, 22Rv1, C4-2, PC3 and DU145 cells after 72 h treatment with 1 µM JQ1 or SP-2509 alone, or in combination. Cell death was assessed using propidium iodide (PI) staining (**A**) or apoptosis was assessed using Annexin-V and PI staining (**B**) followed by FACS analysis. A representative image is shown.

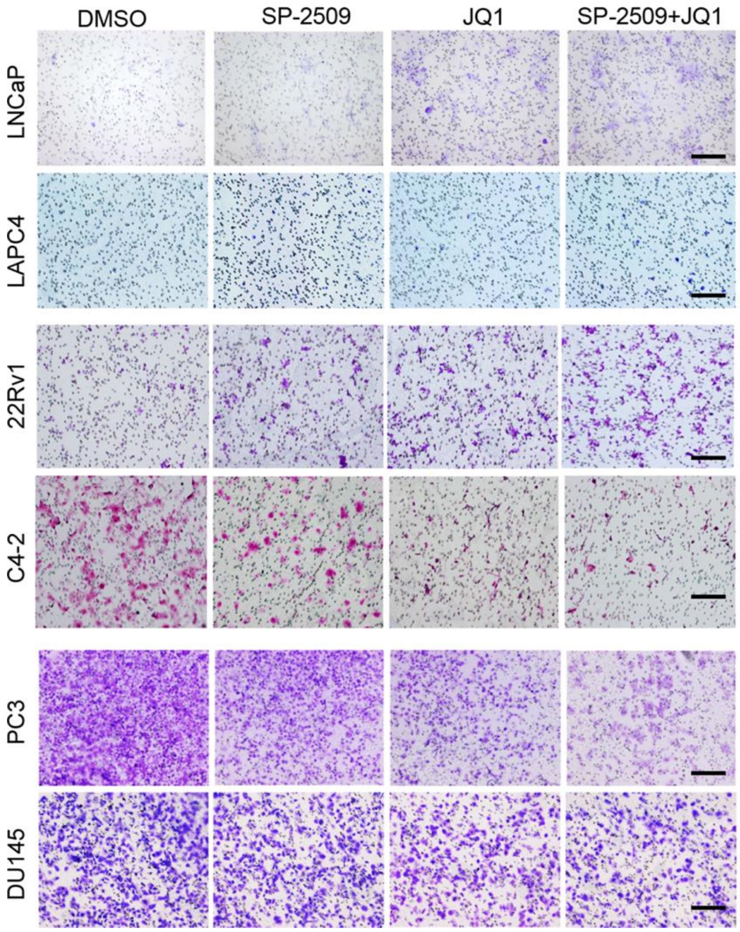

Supplementary Figure 2. The cell invasion analysis was performed after treatment. Representative images are show.

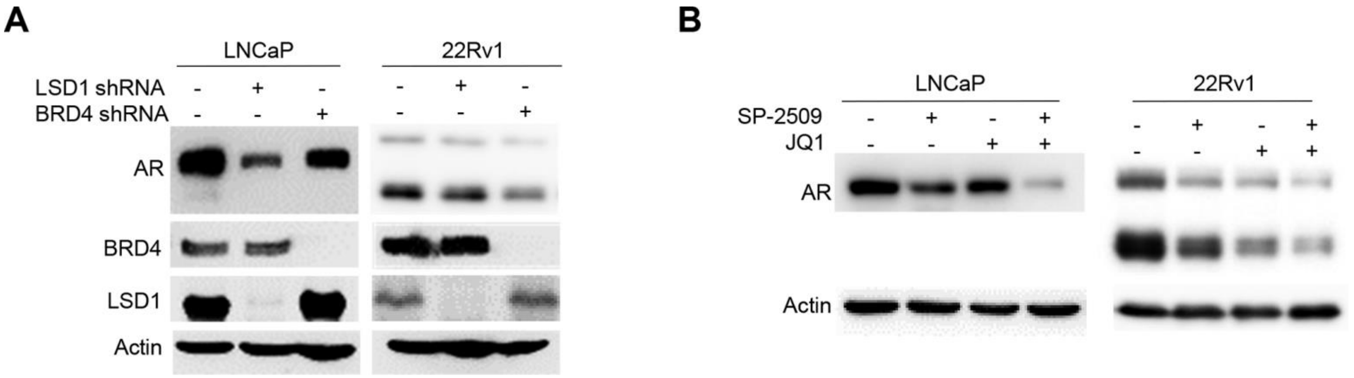

Supplementary Figure 3. Inhibition of LSD1 or BRD4 reduces AR expression. (**A**) knock-down of LSD1 or BRD4 and western blot analysis for AR expression. (**B**) Cells were treated with 1 µM JQ1 or SP-2509 alone, or in combination for 72 h. Total protein lysates were analyzed by immunoblot using the indicated antibodies.
